# Supportive care and in-hospital mortality among children with suspected sepsis in Bamako, Mali

**DOI:** 10.3389/fped.2026.1924848

**Published:** 2026-09-23

**Authors:** William L. Still, Adama Mamby Keita, Milagritos D. Tapia, Sumanth Cherukumilli, Yuanyuan Liang, Kristen A. Stafford, Uma Onwuchekwa, Jane Juma, Samba O. Sow, Karen L. Kotloff, Adnan Bhutta

**Affiliations:** 1 Department of Epidemiology and Public Health, University of Maryland Baltimore, Baltimore, MD, United States; 2 Centre Pour le Développement des Vaccins—Mali, Bamako, Mali; 3 Department of Pediatrics, Division of Pediatric Infectious Diseases and Tropical Pediatrics, University of Maryland School of Medicine, Baltimore, MD, United States; 4 Center for Vaccine Development and Global Health, University of Maryland School of Medicine, Baltimore, MD, United States; 5 Center for International Health, Education, and Biosecurity, Institute of Human Virology, University of Maryland School of Medicine, Baltimore, MD, United States; 6 Department of Pediatrics, Indiana University School of Medicine and Riley Children’s Health, Indianapolis, IN, United States

**Keywords:** antimicrobial resistance, invasive bacterial infection, low- and middle-income countries, Mali, pediatric sepsis, supportive care

## Abstract

**Introduction:**

Sepsis causes significant pediatric hospitalizations and deaths globally. The impact of supportive care interventions remains under-studied in sub-Saharan Africa, where mortality is greatest.

**Methods:**

We conducted a prospective cohort study of children under five years hospitalized with study-defined suspected invasive bacterial infection and ≥2 adapted systemic inflammatory response syndrome (SIRS) criteria at l'Hôpital Gabriel Touré (HGT), a tertiary care center in Bamako, Mali, between January 2021 and July 2024. Associations between supportive care therapies (antibiotics, intravenous fluids, and supplemental oxygen) and in-hospital mortality were evaluated using multivariable logistic regression and time-varying Cox proportional hazards models adjusted for prespecified demographic and clinical covariates.

**Results:**

Among 1,569 enrolled children, 165 (10.5%) had a culture positive for a putative pathogen; 516 (32.9%) died during hospitalization. Most patients received antibiotics (86.4%), while 56.0% received intravenous fluids and 28.7% received supplemental oxygen. Antibiotics and intravenous fluids were not significantly associated with mortality after adjustment. Receipt of supplemental oxygen was associated with higher odds of in-hospital death (aOR: 2.09, 95% CI: 1.50, 2.91), an association potentially reflecting confounding by indication due to greater illness severity among children receiving oxygen.

**Conclusions:**

In this observational cohort with high in-hospital mortality from suspected pediatric sepsis, supportive care therapies were not associated with improved survival after multivariable adjustment. These findings may reflect confounding by indication and a high burden of antimicrobial resistance that may limit the effectiveness of empiric antibiotic therapy. Reducing pediatric sepsis mortality in resource-limited settings will require improved access to microbiologically active antibiotics alongside context-appropriate pediatric critical care interventions.

## 1 Introduction

Infections leading to sepsis are among the most common causes of death worldwide, contributing to over half of all under-five mortality globally (1). Most sepsis cases and deaths occur in low- and middle-income countries (LMICs) (2). Factors likely to contribute to the high burden of sepsis in LMICs include co-morbid conditions such as HIV and severe malnutrition, suboptimal hygiene and vaccination coverage, and fragile healthcare infrastructure (3).

An important step in measuring the burden of sepsis and implementing effective counter-measures in LMICs is to develop a harmonized definition. The 2005 International Pediatric Sepsis Consensus Conference established criteria for diagnosis of sepsis based on expert opinion as suspected or confirmed infection in the presence of systemic inflammatory response syndrome (SIRS); organ dysfunction defined severe sepsis cardiovascular compromise defined septic shock (4). In 2024, the Society of Critical Care Medicine (SCCM) Pediatric Sepsis Definition Task Force launched an effort to develop data-driven criteria that address shortcomings of the 2005 definitions, especially regarding the precision of the definition and its applicability to LMICs (5, 6). Using statistical modeling, SSCM analyzed a retrospective multinational database comprising over 3.0 million pediatric encounters to develop and validate organ dysfunction-based sepsis scores that predict in-hospital pediatric mortality. The resultant Phoenix Sepsis Score defined pediatric sepsis as a suspected infection with a score of at least 2 points, with septic shock defined as the addition of one or more cardiovascular points. Mortality approached 30% among children in LMICs meeting Phoenix criteria for sepsis, and exceeded 30% among those with septic shock, levels estimated to be at least 4-fold greater than that seen in higher resource settings (6).

The need for prospectively collected data from LMICs to validate these findings is recognized (7). However, the application of the Phoenix definition in LMIC settings is greatly constrained by missing data due to the limited availability of trained staff, equipment, and laboratory facilities (8, 9). Thus, the earlier 2005 definition remains the more pragmatic approach for conducting surveillance, research and quality improvement in resource-limited settings, provided that the limitations of the 2005 definitions are explicitly acknowledged.

Despite numerous international efforts to define pediatric sepsis in LMICs, there is a paucity of data describing the effectiveness of clinical management strategies in these settings. The Surviving Sepsis Campaign (SSC) has released guidelines for the management of severe sepsis and septic shock (10), which in an implementation study were shown to improve the quality of sepsis care and associated hospital mortality rates in high-resource settings (11). Recommended supportive care strategies for children in LMICs with suspected sepsis often include antibiotics, intravenous fluids, and supplemental oxygen. Evidence-based guidelines are urgently needed, particularly in LMICs with pediatric populations suffering from high mortality rates, to inform clinical management strategies and guide future efforts to strengthen pediatric sepsis care.

The under-five mortality rate in Mali, a landlocked country in West Africa, ranks among the ten highest globally, estimated at 87 deaths per 1,000 live births in 2024 (12). Although data on pediatric sepsis in Mali are limited, a recent study reported a 21% prevalence of neonatal sepsis among neonates presenting to selected health facilities in Bamako and the Koulikoro region (13). The Centre pour le Développement des Vaccins in Mali (CVD-Mali) is an organization within the Ministry of Health dedicated to public health research and implementation, with a focus on reducing infant and child mortality. Since 2002, CVD-Mali has conducted surveillance to identify the etiology of invasive bacterial infections (IBI) among children aged ≤15 years seeking emergency care at l'Hôpital Gabriel Touré (HGT), the primary pediatric referral hospital located in Bamako. IBI is defined as high fever (axillary temperature ≥ 39 °C) and/or clinician suspicion of IBI, as previously described (14). Despite the substantial burden of pediatric sepsis in sub-Saharan Africa, there are limited prospective data describing the clinical presentation, supportive care management, and outcomes of children with suspected sepsis in routine hospital settings, particularly in Mali. To address this gap, the surveillance protocol was expanded in January 2021, with the objective of characterizing the clinical presentation management, and outcome of systematically-defined suspected sepsis among children under 5 years of age to better inform clinical management strategies and guide future efforts to strengthen pediatric sepsis care. Herein we report the findings from the expanded study.

## 2 Methods

### 2.1 Study design and population

Between January 2021 and July 2024, all children under 5 years of age enrolled in the IBI surveillance study were screened for systemic inflammatory response syndrome (SIRS) criteria. Those with ≥2 SIRS criteria were classified *a priori* as the Sepsis Cohort (SC) and comprise the expanded prospective observational analytic cohort for the present study. SC children were enrolled consecutively throughout the study period, subject to informed consent and the availability of study staff and laboratory resources. Their clinical status was followed until hospital discharge or in-hospital death. All children in the SC underwent clinical data collection detailing the use and timing of supportive care therapies, including antibiotic administration, intravenous fluid therapy, and supplemental oxygen.

SIRS criteria were adapted from international pediatric consensus definitions (4) for use in resource-limited settings (15) and included: (1) axillary temperature of >38.5 °C or <36 °C; (2) abnormal heart rate for age; (3) tachypnea for age; and (4) one or more danger signs (lethargy, irritability, unconscious state, inability to feed, vomiting everything, or convulsive state), as judged by the treating clinician and adapted from the World Health Organization (WHO) Integrated Management of Childhood Illness (IMCI) Handbook (16).

### 2.2 Data collection and variable definitions

Enrolled patients underwent blood culture, with additional specimens from normally sterile sites collected at the clinician's discretion. Blood specimens were inoculated into BACTEC Culture Vials Peds Plus/F flasks (Becton Dickinson, Sparks, MD), stored at room temperature, and transported to the HGT bacteriology laboratory. Samples were incubated in an automated blood culture system (BACTEC FX 40, Becton Dickinson). Bacteria were identified by Gram stain, culture, and biochemical testing, and antibiotic susceptibility was assessed by disc-diffusion methods. Organisms considered contaminants included Gram-positive rods and coagulase-negative staphylococci. Clinically significant organisms isolated on culture were reported to the treating clinician. A thick blood smear was examined for malaria parasitemia.

Administration of supportive care therapies, including antibiotics, intravenous fluids, and supplemental oxygen, was recorded with timing of administration. The name of each systemic antibiotic and the date and time of administration were documented. Intravenous fluid administration was defined as receipt of any intravenous fluid, whereas fluid bolus administration was defined as receipt of intravenous fluids meeting or exceeding the child's calculated Holliday-Segar maintenance fluid requirement within the first 6 h. Oxygen therapy was defined as receipt of supplemental oxygen delivered by nasal cannula. Clinical variables that were documented included anthropometric measures, vital signs, and indicators of severe sepsis. Severe sepsis was defined by any evidence of organ dysfunction (15), including decreased capillary refill, respiratory distress, decreased oxygen saturation (<90% with or without oxygen), acute oliguria, scleral icterus, petechiae/ecchymoses/purpura, or altered consciousness (Blantyre coma score < 5). Clinical variable definitions, including SIRS criteria, severe sepsis indicators, and malnutrition status based on WHO criteria (17), are provided in detail in Appendix 1.

Investigators at HGT collected study data at presentation to the emergency department and throughout hospitalization using REDCap mobile tools (18–20). The study protocol was approved by the Institutional Review Board at the University of Maryland, Baltimore, and the Ethics Committee of University of Sciences, Techniques and Technologies of Bamako (USTTB).

### 2.3 Statistical methods

Descriptive statistics summarize demographic and clinical characteristics. Because this was an exploratory observational analysis embedded within an ongoing surveillance program, no formal sample size calculation was performed. Instead, all consecutively enrolled eligible children meeting the study criteria during the surveillance period were included in the analysis. Associations with in-hospital mortality were assessed using chi-square tests (*α* = 0.05). Primary analyses evaluated associations between supportive therapies (antibiotics, intravenous fluids, supplemental oxygen) and mortality. Exposures were analyzed both as binary variables and stratified by timing of administration (≤60 min, 61–180 min, >180 min, none). Multivariable logistic regression models estimated adjusted odds ratios (aORs) and corresponding 95% confidence intervals. Effect measure modification was assessed for prespecified covariates; interaction terms were retained if *p* < 0.01. Seven prespecified logistic regression analyses evaluated complementary definitions of supportive care exposure, including binary exposure, timing of intervention receipt, and clinically relevant subgroups (Appendix 2). Covariates were selected *a priori* based on clinical relevance and data availability; the complete set of prespecified adjustment covariates included in all adjusted models is provided in Appendix 3. Multicollinearity among adjustment covariates was assessed using variance inflation factors.

Because supportive care therapies were initiated after hospital admission and treatment timing varied across patients, secondary analyses were performed using Cox proportional hazards models with time-varying exposures. These analyses accounted for changes in treatment status over follow-up, reducing bias associated with treating therapies as fixed baseline exposures. Adjusted models included the same prespecified covariates as the logistic regression analyses to account for measured differences in baseline illness severity that may have influenced treatment decisions. Hazard ratios and corresponding 95% confidence intervals were generated using robust standard errors. For each supportive care therapy (antibiotics, intravenous fluids, and supplemental oxygen), the hazard of death among treated patients was compared with that among untreated patients to estimate mortality risk within 48 h and 7 days. The proportional hazards assumption was assessed using the global test and Schoenfeld residual plots. Patients whose discharge or death time was not observed within the analysis window were censored at the end of follow-up for that analysis.

All descriptive statistics, chi-square tests, and multivariable logistic regression analyses were performed using SAS version 9.4 (SAS Institute Inc., Cary, NC, USA). Time-varying Cox proportional hazards survival analyses were performed using Stata/SE version 17 (StataCorp LLC, College Station, TX, USA).

## 3 Results

### 3.1 Study subjects and clinical presentation

Between 15 January 2021 and 31 July 2024, 27,408 children under five years of age were screened in the HGT Emergency Department, of whom 11,397 were hospitalized (Figure 1). Among hospitalized children, 2,420 met eligibility criteria for enrollment in the IBI study. After exclusion due to refusal of consent, staffing or laboratory constraints, death prior to enrollment, or other enrollment limitations, 2,127 were enrolled in the IBI study. Of these, 1,658 met criteria for suspected IBI with ≥2 SIRS criteria. After excluding 89 children due to loss to follow-up without outcome data at discharge, or incomplete case report forms, the final Sepsis Cohort (SC) comprised 1,569 children.

**Figure 1:**
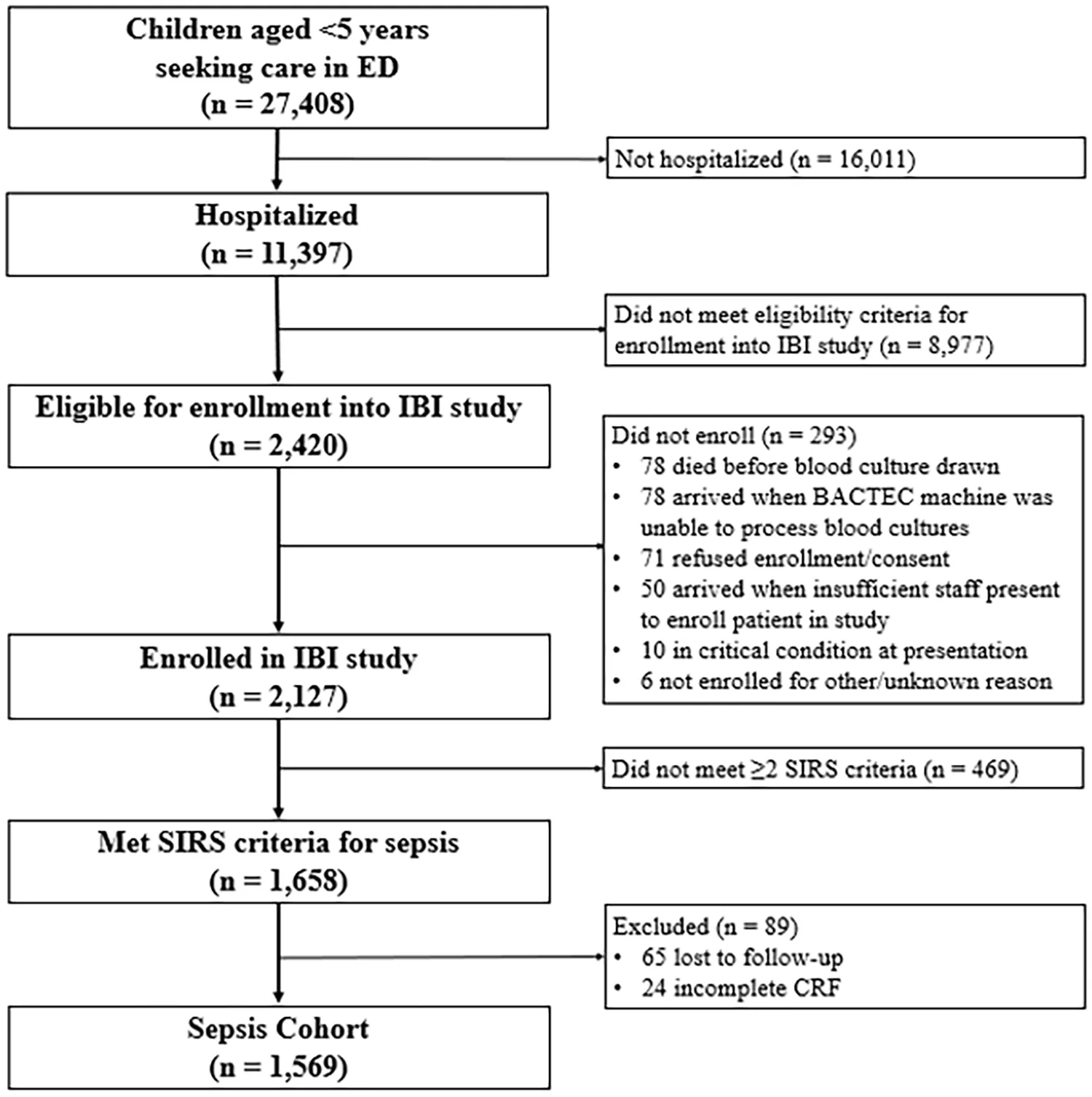
Study flow of participants.

Demographic and clinical characteristics are summarized in Table 1. Most patients were male (54.0%) and non-residents of Bamako (50.6%). Nearly half (49.0%) were aged 12–59 months, 39.8% were aged 29 days—11 months, 4.8% were aged 7–28 days, and 6.4% were neonates aged younger than 7 days. Among children fulfilling SIRS criteria, 54.0% had abnormal temperature (one of whom had hypothermia, temperature < 35 °C, 0.06%), 35.8% had an abnormal heart rate for age, 94.1% had tachypnea for age, and 82.3% had at least one danger sign at presentation. The most common danger signs were recent convulsions (50.3%), lethargy (37.7%), and inability to feed (33.1%).

**Table 1:** Demographic characteristics and presentation on admission among all screened patients and the sepsis cohort.

Characteristic	Sepsis Cohort (col %)
Age group	
Less than seven days	101 (6.4)
7–28 days	75 (4.8)
29 days—11 months	625 (39.8)
12–59 months	768 (49.0)
Sex	
Male	847 (54.0)
Female	722 (46.0)
Bamako residence	
Resident	775 (49.4)
Non-resident	794 (50.6)
SIRS/Sepsis criteria	
Abnormal temperature	848 (54.0)
Abnormal heart rate for age	562 (35.8)
Tachypnea for age	1,476 (94.1)
One or more danger sign	1,292 (82.3)
Irritable	260 (16.6)
Lethargic	592 (37.7)
Unconscious	73 (4.7)
Unable to feed	519 (33.1)
Vomiting everything	151 (9.6)
Convulsions	789 (50.3)
Suspicion of bacterial infection	1,569 (100)
Met criteria for sepsis	1,569 (100)
Total	1,569

A total of 165 patients (10.5%) had a blood, cerebrospinal fluid, or other sterile site culture positive for a putative pathogen (Table 2), including 144 with positive blood cultures. The most frequently isolated pathogens were *Staphylococcus aureus* (*n* = 29), *Klebsiella pneumoniae* (*n* = 25), *Streptococcus pneumoniae* (*n* = 23), *Escherichia* spp. (*n* = 14), and *Pseudomonas aeruginosa* (*n* = 14). Severe acute malnutrition (SAM) was present in 37.1% of children, while 18.1% met criteria for moderate acute malnutrition (MAM) by weight-for-height score. Most patients (73.6%) met criteria for severe sepsis; the most common indicators were Blantyre Coma Score < 5 (56.6%), oxygen saturation <90% (26.3%), and acute oliguria (14.2%). Malaria smear was positive in 27.3% of patients, although only 56.8% of smear-positive children were reported to have received antimalarial therapy. Children with positive malaria smears were significantly more likely to be older (particularly 12–59 months), reside outside Bamako, present with convulsions, and present unconscious (Supplementary Table 1).

**Table 2:** Clinical characteristics and associations with in-hospital mortality among the sepsis cohort.

Characteristic	Characteristic distribution (*N* = 1,569)	Mortality comparison of 1,053 children who survived vs. 516 who died at HGT		
	N (col %)*	Survived (row %)*	Died at HGT (row %)*	*p*-value^†^
Age group				
Less than seven days	101 (6.4)	57 (56.4)	44 (43.6)	.0226
7–28 days	75 (4.8)	43 (57.3)	32 (42.7)	
29 days—11 months	625 (39.8)	426 (68.2)	199 (31.8)	
12–59 months	768 (48.9)	527 (68.6)	241 (31.4)	
Sex				
Male	847 (54.0)	558 (65.9)	289 (34.1)	.2601
Female	722 (46.0)	495 (68.6)	227 (31.4)	
Bamako residence				
Resident	775 (49.4)	526 (67.9)	249 (32.1)	.5277
Non-resident	794 (50.6)	527 (66.4)	267 (33.6)	
SIRS criteria				
Abnormal temperature	848 (54.0)	544 (64.2)	304 (35.8)	.0068
Abnormal heart rate for age	562 (35.8)	378 (67.3)	184 (32.7)	.9262
Tachypnea for age	1,476 (94.1)	994 (67.3)	482 (32.7)	.4371
One or more danger signs	1,292 (82.3)	844 (65.3)	448 (34.7)	.0011
Irritable	260 (16.6)	190 (73.1)	70 (26.9)	.0250
Lethargic	592 (37.7)	356 (60.1)	236 (39.9)	<.0001
Unconscious	73 (4.7)	42 (57.5)	31 (42.5)	.0744
Unable to feed	519 (33.1)	288 (55.5)	231 (44.5)	<.0001
Vomiting everything	151 (9.6)	100 (66.2)	51 (33.8)	.8071
Convulsions	789 (50.3)	531 (67.3)	258 (32.7)	.8736
Diagnosis at admission				
Meningitis	759 (48.4)	492 (64.8)	267 (35.2)	.0615
Pneumonia	417 (26.6)	294 (70.5)	123 (29.5)	.0854
Status prior to HGT				
Received antibiotics before HGT	800 (51.0)	521 (65.1)	279 (34.9)	.0874
Referred from healthcare facility^‡^	935 (65.5)	603 (64.5)	332 (35.5)	.0164
Hospitalized before HGT^‡^	240 (16.8)	145 (60.4)	95 (39.6)	.1260
Microbiology of blood, CSF, and sterile site cultures				
Culture positive for a putative pathogen	165 (10.5)	76 (46.1)	89 (53.9)	<.0001
Culture negative for a putative pathogen	1,404 (89.5)	977 (69.6)	427 (30.4)	
Gram-positive bacteria	66 (4.2)	34 (51.5)	32 (48.5)	.0059
*Enterococcus* spp.	11 (0.7)	3 (27.3)	8 (72.7)	.0048
*S. aureus*	29 (1.8)	20 (69.0)	9 (31.0)	.8303
*S. pneumoniae*	23 (1.5)	9 (39.1)	14 (60.9)	.0040
Group A *Streptococcus*	2 (0.1)	1 (50.0)	1 (50.0)	-
*Streptococcus* spp.	2 (0.1)	0 (0.0)	2 (100.0)	-
Gram-negative bacteria	98 (6.2)	45 (45.9)	53 (54.1)	<.0001
Gram-negative Enterobacterales	61 (3.9)	25 (41.0)	36 (59.0)	<.0001
*Enterobacter* spp.	7 (0.4)	4 (57.1)	3 (42.9)	-
*Escherichia* spp.	14 (0.9)	5 (35.7)	9 (64.3)	.0120
*K. pneumoniae*	25 (1.6)	5 (20.0)	20 (80.0)	<.0001
*Klebsiella* spp.	1 (0.1)	0 (0.0)	1 (100.0)	-
*Pantoea* spp.	9 (0.6)	5 (55.6)	4 (44.4)	.4592
*Proteus* spp.	1 (0.1)	0 (0.0)	1 (100.0)	-
Nontyphoidal *Salmonella*	4 (0.3)	4 (100.0)	0 (0.0)	-
*Salmonella* Typhi	2 (0.1)	2 (100.0)	0 (0.0)	-
Gram-negative non-Enterobacterales	38 (2.4)	21 (55.3)	17 (44.7)	.1155
*Acinetobacter* spp.	6 (0.4)	4 (66.7)	2 (33.3)	-
*Aeromonas* spp.	1 (0.1)	1 (100.0)	0 (0.0)	-
Nonfermenting Gram-negative bacillus	3 (0.2)	2 (67.7)	1 (33.3)	-
*Burkholderia* spp.	2 (0.1)	2 (100.0)	0 (0.0)	-
*Chryseobacterium* spp.	1 (0.1)	0 (0.0)	1 (100.0)	-
*Haemphilus influenzae* type b	10 (0.6)	9 (90.0)	1 (10.0)	.1222
*Haemophilus influenzae* non-typable	1 (0.1)	1 (100)	0 (0)	-
*P. aeruginosa*	14 (0.9)	2 (14.3)	12 (85.7)	<.0001
Yeast	3 (0.2)	0 (0)	3 (100.0)	-
Malnutrition†				
Severe Acute Malnutrition	582 (37.1)	363 (62.4)	219 (37.6)	.0082
Moderate Acute Malnutrition	284 (18.1)	198 (69.7)	86 (30.3)	
No Malnutrition	590 (37.6)	420 (71.2)	170 (28.8)	
Unknown malnutrition status	114 (7.3)	72 (63.2)	42 (36.8)	
Acute malnutrition measured by weight-for-height Z-score (WHZ)				
Severe (WHZ < −3)	481 (30.7)	300 (62.4)	181 (37.6)	.0136
Moderate (−3 ≤ WHZ < −2)	219 (14.0)	156 (71.2)	63 (28.8)	
Mild (WHZ ≥ −2)	746 (47.5)	521 (69.8)	225 (30.2)	
Unknown WHZ	123 (7.8)	76 (61.8)	47 (38.2)	
Acute malnutrition measured by mid-upper arm circumference (MUAC) (among children aged ≥ 6 months only, *n* = 1,108)				
Severe (MUAC < 11.5 cm)	252 (22.7)	156 (61.9)	96 (38.1)	.1675
Moderate (11.5 cm ≤ MUAC < 12.5) cm	247 (22.3)	168 (68.0)	79 (32.0)	
MUAC ≥ 12.5 cm	521 (47.0)	361 (69.3)	160 (30.7)	
Unknown	88 (7.9)	63 (71.6)	25 (28.4)	
Bilateral pitting edema	3 (0.2)	0 (0.0)	3 (100.0)	-
Malaria Status				
Smear +, antimalarials given	243 (15.5)	182 (74.9)	61 (25.1)	.0026
Smear +, no antimalarials given	185 (11.8)	132 (71.4)	53 (28.6)	
Smear −, antimalarials given	248 (15.8)	174 (70.2)	74 (29.8)	
Smear −, no antimalarials given	887 (56.5)	563 (63.5)	324 (36.5)	
No smear, antimalarials given	1 (0.1)	0 (0.0)	1 (100.0)	
No smear, no antimalarials given	5 (0.3)	2 (40.0)	3 (60.0)	
Severe sepsis indicators				
Met criteria for severe sepsis	1,154 (73.6)	711 (61.6)	443 (38.4)	<.0001
Blantyre Coma Score < 5	888 (56.6)	528 (59.5)	360 (40.5)	<.0001
Oxygen saturation < 90%	413 (26.3)	240 (58.1)	173 (41.9)	<.0001
Acute oliguria	223 (14.2)	140 (62.8)	83 (37.2)	.1371
Signs of respiratory distress	15 (1.0)	11 (73.3)	4 (26.7)	.8504
Peripheral or central cyanosis	9 (0.6)	4 (44.4)	5 (55.6)	.1466
Decreased capillary refill	8 (0.5)	5 (62.5)	3 (37.5)	.5126
Petechiae, ecchymoses, or purpura	3 (0.2)	3 (100.0)	0 (0.0)	-
Scleral icterus	0 (0.0)	0 (0.0)	0 (0.0)	-
Length of stay				
<1 day	156 (9.9)	34 (21.8)	122 (78.2)	<.0001
1 day	266 (17.0)	84 (31.6)	182 (68.4)	
2 days	163 (10.4)	85 (52.1)	78 (47.9)	
3 days	144 (9.2)	101 (70.1)	43 (29.9)	
4–5 days	234 (14.9)	206 (88.0)	28 (12.0)	
6–7 days	200 (12.7)	173 (86.5)	27 (13.5)	
8–10 days	157 (10.0)	146 (93.0)	11 (7.0)	
11–14 days	101 (6.4)	92 (91.1)	9 (8.9)	
15+ days	148 (9.4)	132 (89.2)	16 (10.8)	
Total	1,569	1,053 (67.1)	516 (32.9)	

* Data are presented as *n* (%). Column 2 presents the distribution of each characteristic in the Sepsis Cohort (percentage of total cohort, *N* = 1,569). For Columns 3 and 4, percentages represent row percentages within each level of the characteristic, such that values indicate the proportion of individuals in each characteristic category who survived or died.

† *p*-values were calculated using Pearson's chi-square test for categorical variables. *p*-values were not reported for comparisons involving extremely sparse categories (e.g., rare binary variables or low-frequency pathogen groups), where results were not considered interpretable due to limited sample size; these are indicated as not estimable (-).

‡ Among patients who were assessed for referral from other healthcare facility prior to arrival at HGT (*n* = 1,428).

### 3.2 Mortality

Among 1,569 children in the SC, 516 (32.9%) died during hospitalization and 1,053 (67.1%) survived to discharge. Mortality was not associated with sex or Bamako residence but differed significantly by age, with the highest case fatality ratio (CFR) observed among neonates aged <7 days (43.6%) and 7–28 days (42.7%) (Table 2).

Children presenting with at least one danger sign had a higher mortality than those without any danger signs (34.7% vs. 24.5%, *p* = 0.011) (Table 2). Lethargy (39.9%, *p* < 0.0001) and inability to feed (44.5%, *p* < 0.0001) were strongly associated with mortality. Mortality was also higher among children with positive sterile-site cultures compared with culture-negative children (53.9% vs. 30.4%, *p* < 0.0001). Pathogens with the highest CFRs were *P. aeruginosa* (85.7%), *K. pneumoniae* (80.0%), *Enterococcus* spp. (72.7%), *Escherichia* spp. (64.3%), and *S. pneumoniae* (60.9%).

In-hospital mortality differed significantly by nutritional status and was highest among children with SAM (38.3%), followed by those with MAM (30.3%) (*p* = 0.0082) (Table 2). When WHZ and MUAC classifications were analyzed separately, mortality was significantly associated with WHZ (*p* = 0.0136) but not MUAC (*p* = 0.1675). Children meeting criteria for severe sepsis also had higher mortality than those without severe sepsis (38.4% vs. 17.6%, *p* < 0.0001), particularly among patients with oxygen saturation < 90% (41.9%) or Blantyre Coma Score < 5 (40.5%). Mortality was highest among children hospitalized for less than one day (78.2%) and decreased with increasing length of stay (*p* < 0.0001). Associations between clinical characteristics and in-hospital mortality stratified by malaria smear status are presented in Supplementary Table 2. Overall, the pattern of associations was similar among children with and without malaria.

Among children with culture-confirmed bacterial infection who received antibiotics and had corresponding susceptibility data, mortality differed according to ceftriaxone susceptibility but not gentamicin susceptibility (Supplementary Table 3). None of the eight children who received ceftriaxone for a ceftriaxone-susceptible isolate died, compared with 17 of 23 (73.9%) whose isolates were ceftriaxone-resistant (*p* = 0.0004). In contrast, mortality was similar among children with gentamicin-susceptible (15/31, 48.4%) and gentamicin-resistant isolates (23/40, 57.5%; *p* = 0.48). Additionally, among the 38 children infected with Enterobacterales species and treated with empiric therapy (either ceftriaxone, cefotaxime, or ciprofloxacin), 33 were resistant (86.8%) to the therapy administered. An additional 17 children infected with the predominant non-Enterobacterales pathogens (*Acinetobacter* or *Pseudomonas*) were treated with ceftriaxone or cefotaxime, neither of which has reliable activity, suggesting that over 90% of children overall with these Gram-negative infections received an antibiotic to which their treatment was likely resistant. Mortality was comparable among those with susceptible (60%) and resistant (68%) infections.

### 3.3 Multivariable logistic regression analysis

Crude and adjusted logistic regression models are presented in Table 3. No covariates met the prespecified criteria for effect measure modification.

**Table 3:** Associations between supportive care therapies (antibiotics, fluids, and supplemental oxygen) and in-hospital mortality. Crude and adjusted odds ratios are provided with corresponding *p*-values.

	N (col %)	Survived (row %)	Deceased (row %)	Crude OR (95% CI)	Crude *p*-value	Adjusted OR* (95% CI)	Adjusted *p*-value
Analysis 1—Binary exposure (among all children in the Sepsis Cohort, *n* = 1,569)							
Received antibiotics	1,355 (86.4)	890 (65.7)	465 (34.3)	1.67 (1.20, 2.33)	.0026	1.40 (.90, 2.18)	.1411
No antibiotics (ref)	214 (13.6)	163 (76.2)	51 (23.8)	(ref)	-	(ref)	-
Received fluids	878 (56.0)	562 (64.0)	316 (36.0)	1.38 (1.11, 1.71)	.0032	1.27 (.97, 1.65)	.0802
No fluids (ref)	691 (44.0)	491 (71.1)	200 (28.9)	(ref)	-	(ref)	-
Received oxygen	451 (28.7)	250 (55.4)	201 (44.6)	2.05 (1.63, 2.57)	<.0001	2.09 (1.50, 2.91)	<.0001
No oxygen (ref)	1,118 (71.3)	803 (71.8)	315 (28.2)	(ref)	-	(ref)	-
Analysis 2—Timing of intervention (among children with known time of intervention receipt or no receipt: antibiotics *n* = 1,549; fluids *n* = 1,564; oxygen *n* = 1,533)							
Antibiotics ≤ 1 h	606 (38.6)	398 (65.7)	208 (34.3)	1.67 (1.17, 2.38)	.0054	1.33 (.81, 2.17)	.2592
Antibiotics 1–3 h	531 (33.8)	343 (64.6)	188 (35.4)	1.75 (1.22, 2.51)	.0024	1.48 (.90, 2.44)	.1233
Antibiotics > 3 h	198 (12.6)	131 (66.2)	67 (33.8)	1.63 (1.06, 2.51)	.0255	1.72 (.93, 3.19)	.0849
No antibiotics (ref)	214 (13.6)	163 (76.2)	51 (23.8)	(ref)	-	(ref)	-
Fluids ≤ 1 h	374 (23.8)	235 (62.8)	139 (37.2)	1.45 (1.11, 1.90)	.0061	1.31 (.91, 1.89)	.1455
Fluids 1–3 h	349 (22.2)	224 (64.2)	125 (35.8)	1.37 (1.04, 1.80)	.0242	1.32 (.90, 1.92)	.1548
Fluids > 3 h	150 (9.6)	100 (66.7)	50 (33.3)	1.23 (.84, 1.79)	.2868	1.17 (.69, 2.00)	.5625
No fluids (ref)	691 (44.0)	491 (71.1)	200 (28.9)	(ref)	-	(ref)	-
Oxygen ≤ 1 h	330 (21.0)	181 (54.8)	149 (45.2)	2.10 (1.63, 2.70)	<.0001	2.12 (1.46, 3.06)	<.0001
Oxygen 1–3 h	73 (4.7)	40 (54.8)	33 (45.2)	2.10 (1.30, 3.40)	.0023	2.07 (1.12, 3.84)	.0204
Oxygen > 3 h	48 (3.1)	29 (60.4)	19 (39.6)	1.67 (.92, 3.02)	.0900	1.67 (0.76, 3.67)	.2044
No oxygen (ref)	1,118 (71.3)	803 (71.8)	315 (28.2)	(ref)	-	(ref)	-
Analysis 3—Antibiotic timeliness (among treated children with known time of receipt, *n* = 1,335)							
Antibiotics ≤ 1 h	606 (45.4)	398 (65.7)	208 (34.3)	0.97 (.78, 1.22)	.8021	0.88 (.67, 1.16)	.3730
Antibiotics > 1 h (ref)	729 (54.6)	474 (65.0)	255 (35.0)	(ref)	-	(ref)	-
Analysis 4—Fluid timeliness (among treated children with known time of receipt, *n* = 873)							
Fluids ≤ 1 h	374 (42.8)	235 (62.8)	139 (37.2)	1.10 (.83, 1.45)	.5232	0.96 (.68, 1.36)	.8188
Fluids > 1 h (ref)	499 (57.2)	324 (64.9)	175 (35.1)	(ref)	-	(ref)	-
Analysis 5—Oxygen timeliness (among treated children with known time of receipt, *n* = 451)							
Oxygen ≤ 1 h	330 (73.2)	181 (54.8)	149 (45.2)	1.09 (.72, 1.66)	.6811	1.05 (.63, 1.76)	.8466
Oxygen > 1 h (ref)	121 (26.8)	69 (57.0)	52 (43.0)	(ref)	-	(ref)	-
Analysis 6—Oxygen among children with an indication (among children with oxygen saturation on room air ≤ 90%, have chest indrawing, or have difficulty breathing, *n* = 424)							
Received oxygen	287 (67.7)	164 (57.1)	123 (42.9)	1.15 (.76, 1.75)	.5017	1.83 (1.06, 3.16)	.0303
No oxygen (ref)	137 (32.3)	83 (60.6)	54 (39.4)	(ref)	-	(ref)	-
Analysis 7—Maintenance and bolus fluids (among all children, *n* = 1,569)							
Maintenance fluids	843 (53.7)	540 (64.1)	303 (35.9)	1.38 (1.11, 1.71)	.0037	1.26 (.97, 1.65)	.0875
Bolus fluids	35 (2.2)	22 (62.9)	13 (37.1)	1.45 (.72, 2.94)	.3011	1.47 (.60, 3.41)	.4245
No fluids (ref)	691 (44.0)	491 (71.1)	200 (28.9)	(ref)	-	(ref)	-

* Adjusted for prespecified demographic and clinical covariates (Appendix 3): age category, malnutrition status, malaria smear result and antimalarial treatment, pathogen isolated on culture, pneumonia diagnosis at admission, meningitis diagnosis at admission, abnormal temperature, abnormal heart rate, tachypnea, irritability, lethargy, unconsciousness, inability to feed, convulsions, low Blantyre Coma Score, low oxygen saturation, inability to urinate, duration of hospitalization, and receipt of antibiotics prior to hospital admission.

Most patients (86.4%) received antibiotics during hospitalization; 38.6% received antibiotics within one hour of arrival. After adjustment, antibiotic administration was not significantly associated with in-hospital mortality [adjusted OR (aOR): 1.40, 95% CI: 0.90–2.18], including when administered within one hour of arrival (aOR: 0.88, 95% CI: 0.67–1.16).

Overall, 56.0% of patients received fluid therapy, including 23.8% within one hour of arrival. Fluid administration was not significantly associated with mortality (aOR: 1.27, 95% CI: 0.97–1.65), and timing of fluid administration was not associated with outcome. Few patients (2.2%) received fluid boluses, limiting the precision of this analysis. Bolus administration was not significantly associated with mortality (aOR: 1.47, 95% CI: 0.60–3.41).

Supplemental oxygen therapy via nasal cannula was administered to 28.7% of patients, including 21.0% within one hour of arrival. Oxygen therapy was associated with higher odds of in-hospital mortality (aOR: 2.09, 95% CI: 1.50–2.91). Among patients meeting criteria for oxygen therapy indication (oxygen saturation < 90%, chest indrawing, or difficulty breathing), 67.7% received oxygen; oxygen therapy remained associated with increased mortality in this subgroup (aOR: 1.83, 95% CI: 1.06–3.16). No patients received CPAP or other ventilatory support.

### 3.4 Time-varying Cox regression analysis

Among all deaths in the SC, 68.0% (351/516) occurred within 48 h of arrival, and 92.2% (476/516) occurred within 7 days. Children who did not experience in-hospital death during the analysis period were censored at 48 h (1,218/1,569, 77.6%) or 7 days (1,093/1,569, 69.7%).

In adjusted time-varying Cox regression analyses (Table 4), antibiotic administration was associated with increased hazard of death by 48 h [adjusted hazard ratio (aHR): 1.57, 95% CI: 1.11–2.20] and by 7 days (aHR: 1.71, 95% CI: 1.24–2.37) compared with patients who did not receive antibiotics. Fluid therapy was not significantly associated with mortality at either time point. Oxygen therapy was associated with increased hazard of death within 48 h (aHR: 2.12, 95% CI: 1.60–2.88) and within 7 days (aHR: 1.87, 95% CI: 1.44–2.42). There was no evidence of violations of the proportional hazards assumption for the primary exposure variables.

**Table 4:** Crude and adjusted 48 h and 7-day hazard ratios of in-hospital mortality by supportive care measure.

Supportive Care Measure Comparison	Crude HR (95% CI)	*p*-value	Adjusted HR* (95% CI)	*p*-value
Up to 48 h post-enrollment				
Received antibiotics: No antibiotics	1.54 (1.12, 2.11)	.008	1.57 (1.11, 2.20)	.010
Received fluids: No fluids	1.33 (1.07, 1.64)	.010	1.07 (.85, 1.35)	.556
Received oxygen: No oxygen	1.84 (1.48, 2.28)	<.001	2.12 (1.60, 2.88)	<.001
Up to 7 days post-enrollment				
Received antibiotics: No antibiotics	1.75 (1.30, 2.35)	<.001	1.71 (1.24, 2.37)	.001
Received fluids: No fluids	1.33 (1.10, 1.59)	.003	1.11 (.91, 1.35)	.309
Received oxygen: No oxygen	1.75 (1.46, 2.11)	<.001	1.87 (1.44, 2.42)	<.001

* Adjusted for prespecified demographic and clinical covariates (Appendix 3): age category, malnutrition status, malaria smear result and antimalarial treatment, pathogen isolated on culture, pneumonia diagnosis at admission, meningitis diagnosis at admission, abnormal temperature, abnormal heart rate, tachypnea, irritability, lethargy, unconsciousness, inability to feed, convulsions, low Blantyre Coma Score, low oxygen saturation, inability to urinate, duration of hospitalization, and receipt of antibiotics prior to hospital admission.

## 4 Discussion

This study represents one of the largest prospective analyses of suspected pediatric sepsis outcomes in sub-Saharan Africa to date. Although fewer than 10% of children presenting to the emergency department met eligibility criteria for suspected IBI, the majority (78%) of hospitalized children enrolled in the IBI study met the case definition for suspected sepsis (≥2 SIRS criteria). Among these children, nearly one-third died during hospitalization at HGT. Our case-fatality ratio is therefore consistent with the mortality estimates suggested by the Society of Critical Care Medicine (5).

These outcomes from LMICs contrast sharply with those seen in high-income countries, where mortality rates for sepsis are approximately 7.1% (5). Our findings highlight three major factors that may contribute to these disparities. First, the increasing prevalence of multi-resistant organisms in LMICs, particularly among young infants, limits the effectiveness of available empiric antibiotic regimens (21). Our observations of a strong association between antimicrobial resistance and mortality among children treated with ceftriaxone support this hypothesis. Second, optimal delivery of fluid resuscitation for children with SIRS has not been fully elucidated, with prior studies raising concern that fluid boluses can worsen outcomes in some resource-limited settings (22). Third, the only option for respiratory support in our study population was nasal cannula oxygen. Importantly, in many LMIC settings, including HGT, the mechanisms to provide adequate ventilation are lacking, including staffing with round-the-clock trained personnel, a reliable oxygen supply, and delivery devices such as ventilators and continuous positive airway pressure (CPAP) machines.

The absence of an observed overall survival benefit associated with these supportive care measures was unexpected but biologically plausible given the complexity of pediatric sepsis in resource-limited settings. Most patients received antibiotics, while fluid and oxygen therapy were administered less consistently. Neither antibiotic administration, fluid therapy, nor supplemental oxygen delivered via nasal cannula was associated with improved survival. The observed direction of some associations is difficult to interpret in the absence of a randomized controlled trial; however, these findings align with ongoing uncertainty surrounding sepsis management in resource-limited settings. A decade-long study in Uganda with over 10,000 sepsis patient visits found that patients treated with both fluids and antibiotics had a significantly higher risk of death (RR = 1.55, 95% CI: 1.10–2.00) compared to patients without antibiotics and fluids, while observing no impact of fluids alone (23). Additional investigation is needed to better define optimal fluid management strategies for pediatric sepsis in settings where advanced monitoring and critical care support are limited, given the controversy surrounding sepsis guidelines and several studies demonstrating a negative impact of fluids on mortality among patients in sub-Saharan Africa (22, 24). Current SSC guidelines recommend against routine fluid bolus administration in children without hypotension (10); however, blood pressure was not routinely measured at HGT, limiting assessment of guideline-concordant management. Moreover, because relatively few children received fluid boluses, analyses of bolus administration were underpowered and should therefore be considered exploratory.

Antibiotic administration was not significantly associated with increased mortality in logistic regression analysis, but time-varying survival analyses demonstrated an increased hazard of death among patients who received antibiotics. The logistic and Cox regression findings were directionally consistent, although the association reached statistical significance only in the Cox models. Logistic regression estimates the association with mortality over the entire follow-up period without incorporating the timing of death, whereas Cox regression incorporates event times, censoring, and the timing of antibiotic initiation. The Cox analysis is therefore less susceptible to immortal time bias. In addition, the observed positive associations should not be interpreted as harmful effects of antibiotic treatment because confounding by indication is likely: children with more severe illness may have been more likely to receive antibiotics.

Several additional factors likely contributed to the absence of an observed overall survival benefit associated with antibiotic administration in this cohort. First, among children with available susceptibility data, ceftriaxone susceptibility was strongly associated with survival, underscoring the importance of microbiologically active empiric therapy. These findings suggest that the absence of an overall survival benefit should not be interpreted as evidence that antibiotics lack efficacy, but rather reflect the combined challenges of antimicrobial resistance and appropriate empiric antibiotic selection. More than half of Gram-negative non-Enterobacterales isolates were *Acinetobacter* spp. or *Pseudomonas aeruginosa*, organisms that are intrinsically resistant or frequently resistant to the empiric antibiotics most commonly administered in this cohort, particularly ceftriaxone and gentamicin. This mismatch between local pathogen epidemiology and available empiric treatment options represents a major challenge in settings such as Mali, where affordable alternative antibiotics are often not available. Second, only 10.5% of patients had a positive blood, cerebrospinal fluid, or sterile-site culture, suggesting that some children may have succumbed from multi-organ failure or had reached a self-sustaining cascade of cytokine release, complement activation, coagulopathy, endothelial injury, and mitochondrial dysfunction that can continue independent of whether the causative organism is still detectable. Other children may have had non-bacterial etiologies of sepsis. Viral and parasitic infections may have contributed substantially to disease burden in this population. Respiratory syncytial virus (RSV) has a documented burden at HGT (25) and more than one-quarter of patients had a positive malaria smear, many of whom did not receive antimalarial therapy. Third, over half of children were reportedly exposed to antibiotics prior to hospitalization, which may have reduced culture yield and complicated pathogen identification. Together, these findings suggest that the absence of an observed overall survival benefit was more likely driven by antimicrobial resistance, empiric antibiotic mismatch, and the heterogeneous etiologies and pathogenetic manifestations of suspected sepsis than by a lack of antibiotic efficacy.

Supplemental oxygen therapy (delivered by nasal cannula only) was associated with significantly increased odds and hazard of mortality. This finding should be interpreted cautiously, as children receiving oxygen were likely more severely ill at presentation, and residual confounding by illness severity likely remained despite adjustment. Additionally, oxygen delivery at HGT was limited to low-flow nasal cannula, while advanced respiratory support options such as invasive mechanical ventilation and non-invasive positive pressure ventilation were largely unavailable. Children with sepsis-related respiratory failure may therefore require interventions beyond oxygen supplementation alone. Evidence from other studies on the effectiveness of oxygen delivery modalities—including nasal cannula, high-flow nasal cannula, and continuous positive airway pressure (CPAP)—remains limited and inconclusive (26–28); thus, further research is warranted.

The observed associations between supportive care therapies and mortality should be interpreted within the context of the observational study design. Although adjusted models incorporated multiple measures of baseline illness severity and time-varying Cox analyses accounted for changes in treatment status over time, the impact of residual confounding and other unmeasured factors cannot be excluded. Consequently, these analyses identify associations rather than causal effects and should not be interpreted as evidence that supportive care interventions increase mortality or lack clinical benefit.

The definition of suspected sepsis used for this cohort is important. The new pediatric sepsis definition (5) evolved from SIRS-based criteria toward organ dysfunction-based criteria. The Phoenix criteria identify sepsis among children with suspected infection using a Phoenix Sepsis Score of at least 2 points, reflecting respiratory, cardiovascular, coagulation, and/or neurologic dysfunction. These criteria are conceptually preferable for identifying children with life-threatening organ dysfunction and were developed to improve prognostic performance compared with prior SIRS-based criteria (4–6). However, many variables (such as blood pressure, lactate, vasoactive medication use, platelet count, and coagulation profile) required for Phoenix scoring are not routinely available at hospitals in low-resource settings, and concerns have been raised regarding the applicability of newer pediatric sepsis criteria in LMIC settings (9). Our approach therefore used the prior SIRS-based definition and incorporated recommendations adapted for resource-limited environments (15). Although the SIRS-based definition likely included some children without established organ dysfunction, it identified a population at increased risk of adverse outcomes in this setting. The very high mortality, frequent danger signs, hypoxemia, altered mental status, severe malnutrition, and culture-positive invasive bacterial infections indicate that the definition nevertheless captured children with suspected life-threatening infection.

This study has several strengths. The analysis was embedded within a long-standing prospective surveillance platform with standardized enrollment procedures and continuous staffing by trained CVD-Mali investigators, enabling comprehensive data collection with limited missingness. The large sample size and extended surveillance period strengthen the precision of these findings and may support generalizability to similar resource-limited pediatric settings in SSA.

Several limitations should also be considered. First, as with all observational studies, residual confounding by indication and other unmeasured factors cannot be excluded despite adjustment for measured indicators of illness severity and the use of time-varying survival analyses. Second, diagnostic evaluation for organ dysfunction was limited by resource availability, and use of SIRS-based inclusion criteria may have resulted in a sensitive but less specific sepsis definition, particularly given the high prevalence of tachypnea among enrolled patients. Consequently, some children with localized respiratory or other infections who did not have life-threatening organ dysfunction may have been included, potentially diluting observed associations between supportive care interventions and mortality. Third, clinician awareness of study activities may have influenced management decisions, introducing potential Hawthorne effect bias. Fourth, post-discharge outcomes were not assessed, despite evidence that mortality following hospitalization for pediatric infection may be substantial (29, 30). Fifth, children who died prior to enrollment or were too critically ill to enroll were excluded, which may have underestimated overall mortality and biased estimates of treatment associations among the sickest patients. Because many deaths occurred early after presentation, exclusion of these children may have reduced our ability to detect potential benefits of timely supportive care interventions. Sixth, odds ratios were used instead of relative risks due to model convergence limitations, potentially exaggerating effect estimates. Seventh, although only 56.8% of children with positive malaria smears received antimalarial therapy, evaluation of the relationship between antimalarial therapy and mortality was beyond the scope of this study and warrants further investigation. Finally, this study period partially overlapped with the COVID-19 pandemic, and changes in healthcare utilization or hospital capacity during this period may have influenced patient presentation, clinical management, and outcomes. Pandemic-related delays in care-seeking or resource constraints may have resulted in presentation with more advanced illness or affected delivery of supportive care, potentially influencing observed mortality and treatment-outcome associations; data on SARS-CoV-2 coinfection were not available.

## 5 Conclusions

Collectively, these findings highlight the persistently high burden of pediatric sepsis mortality in resource-limited settings. Nearly one in three children under five with study-defined sepsis died during hospitalization despite frequent administration of antibiotics, intravenous fluids, and supplemental oxygen. In this observational analysis, overall antibiotic therapy and fluid administration were not associated with improved survival, while oxygen therapy was associated with increased mortality, an association that may reflect residual confounding by illness severity. These findings emphasize the urgent need for improved pediatric sepsis management strategies adapted to resource-limited settings.

Future efforts should focus on strengthening diagnostic capacity; improving access to effective antibiotics with activity against multidrug-resistant Gram-negative pathogens and ensuring that empiric treatment guidelines reflect local antimicrobial resistance patterns; expanding respiratory support capabilities, including ventilatory support; better understanding of optimal fluid resuscitation for children from LMICs with SIRS; and implementing context-appropriate sepsis care bundles. Prospective interventional studies and implementation research are needed to determine which supportive care strategies most effectively reduce pediatric sepsis mortality in LMIC settings.

## Data Availability

The raw data supporting the conclusions of this article will be made available by the authors, without undue reservation.
