# Supplementary material for: Supportive care and in-hospital mortality among children with suspected sepsis in Bamako, Mali
Source: Front Pediatr. 2026 Sep 23;14:1924848. doi: 10.3389/fped.2026.1924848 (PMC13642601; doi:10.3389/fped.2026.1924848)
Supplement: Supplementary file 1 [file Supplementaryfile1.docx]

**Appendix 1.** Clinical characteristics and definitions.

| **Variable** | **Definition** |
| --- | --- |
| **Systemic inflammatory response syndrome (SIRS)** | Presence of **≥2 of 4 abnormal physiologic criteria**: temperature, heart rate for age, respiratory rate for age, and clinical condition (IMCI danger signs), as defined below |
| **Sepsis** | Suspected invasive bacterial infection **plus ≥2 SIRS criteria** |
| **Severe sepsis** | Suspected invasive bacterial infection **plus ≥2 SIRS criteria**, with evidence of organ dysfunction or severe physiologic derangement (see below) |
| **Temperature (abnormal)** | >38.5°C or <36.0°C |
| **Hypothermia** | <35.0°C |
| **Heart rate (tachycardia)** | <7 days: >180 bpm; 7 days–<1 month: >180 bpm; 1 month–<1 year: >180 bpm; 1–5 years: >140 bpm |
| **Heart rate (bradycardia)** | <7 days: <100 bpm; 7 days–<1 month: <100 bpm; 1 month–<1 year: <90 bpm; not defined for ≥1 year |
| **Respiratory rate (tachypnea)** | <7 days: >50 breaths/min; 7 days–<1 month: >40 breaths/min; 1 month–<1 year: >30 breaths/min; 1–5 years: >22 breaths/min |
| **Clinical condition (IMCI danger signs)** | Any of: irritability, lethargy, unconscious state, inability to feed, vomiting everything, or convulsions within the past 24 hours, as assessed by clinician, per WHO IMCI guidelines (14) |
| **Severe sepsis (organ dysfunction criteria)** | Any of: capillary refill delay; Blantyre Coma Score <5; oxygen saturation <90% on room air; acute oliguria (no urine output in preceding 24 hours); peripheral or central cyanosis; petechiae, ecchymoses, or purpura; scleral icterus; or signs of respiratory distress |
| **Severe acute malnutrition (SAM)** | Any of: weight-for-height Z-score (WHZ) < -3; MUAC <11.5 cm (children ≥6 months); or bilateral pitting edema |
| **Moderate acute malnutrition (MAM)** | Children ≥6 months, not meeting SAM criteria, with WHZ ≥ -3 and < -2 and/or MUAC ≥11.5 cm and <12.5 cm |
| **No acute malnutrition** | WHZ ≥ -2 and MUAC ≥12.5 cm |
| **Unknown nutritional status** | Missing both WHZ and MUAC |

**Appendix 2.**

Seven logistic regression analyses were utilized evaluating the three types of supportive care interventions: antibiotics, fluid therapy, and supplemental oxygen (administered via nasal cannula). **Analysis 1** examined each intervention as a binary exposure (received versus not received) among all children (n = 1569). **Analysis 2** evaluated associations according to timing of intervention receipt (≤60 minutes of arrival, >60 minutes to ≤180 minutes after arrival, >180 minutes after arrival, versus no intervention) among children with known timing of intervention receipt or no receipt of the intervention, including antibiotics (n = 1549), intravenous fluids (n = 1564), and oxygen therapy (n = 1533). **Analyses 3-5** were restricted to children who received the intervention and had a known time of administration, comparing receipt within ≤1 hour versus >1 hour after arrival for antibiotics (n = 1335), intravenous fluids (n = 873), and oxygen therapy (n = 451), respectively. **Analysis 6** evaluated oxygen therapy among children with an indication for oxygen therapy (defined as patients who have an oxygen saturation on room air ≤ 90%, have chest indrawing, or have difficulty breathing) and compared receipt versus non-receipt of oxygen therapy (n = 424). **Analysis 7** compared maintenance intravenous fluids, fluid boluses, and no intravenous fluids among all children (n = 1569).

**Appendix 3.**

The multivariable logistic regression analyses presented in Table 3 and the time-varying Cox proportional hazards analyses presented in Table 4 were adjusted for the same prespecified covariates: age category, malnutrition status, malaria smear result and antimalarial treatment, pathogen isolated on culture, pneumonia diagnosis at admission, meningitis diagnosis at admission, abnormal temperature, abnormal heart rate, tachypnea, irritability, lethargy, unconsciousness, inability to feed, convulsions, low Blantyre Coma Score, low oxygen saturation, inability to urinate, duration of hospitalization, and receipt of antibiotics prior to hospital admission.

**Supplementary Table 1.** Demographic characteristics and presentation on admission among the Sepsis Cohort, stratified by malaria smear positive or smear negative. Six patients with unknown malaria smear status are omitted.

| **Characteristic** | **Sepsis Cohort  (col %) N = 1569** | **Malaria Smear Positive (col %) N = 428** | **Malaria Smear Negative (col %) N = 1135** | **p-value** |
| --- | --- | --- | --- | --- |
| **Age Group** |  |  |  |  |
| Less than seven days | 101 (6.4) | 7 (1.6) | 93 (8.2) | <0.001 |
| 7 – 28 days | 75 (4.8) | 9 (2.1) | 66 (5.8) |  |
| 29 days – 11 months | 625 (39.8) | 136 (31.8) | 484 (42.6) |  |
| 12 – 59 months | 768 (49.0) | 276 (64.5) | 492 (43.3) |  |
| **Sex** |  |  |  |  |
| Male | 847 (54.0) | 228 (53.3) | 618 (54.4) | 0.677 |
| Female | 722 (46.0) | 200 (46.7) | 517 (45.6) |  |
| **Bamako Residence** |  |  |  |  |
| Resident | 775 (49.4) | 186 (43.5) | 585 (51.5) | 0.004 |
| Non-resident | 794 (50.6) | 242 (56.5) | 550 (48.5) |  |
| **SIRS/Sepsis Criteria** |  |  |  |  |
| Abnormal temperature | 848 (54.0) | 209 (48.8) | 636 (56.0) | 0.011 |
| Abnormal heart rate for age | 562 (35.8) | 164 (38.3) | 395 (34.8) | 0.196 |
| Tachypnea for age | 1476 (94.1) | 411 (96.0) | 1059 (93.3) | 0.042 |
| One or more danger signs | 1292 (82.3) | 349 (81.5) | 939 (82.7) | 0.582 |
| Irritable | 260 (16.6) | 58 (13.6) | 201 (17.7) | 0.049 |
| Lethargic | 592 (37.7) | 170 (39.7) | 419 (36.9) | 0.308 |
| Unconscious | 73 (4.7) | 33 (7.7) | 39 (3.4) | <0.001 |
| Unable to feed | 519 (33.1) | 132 (30.8) | 383 (33.7) | 0.276 |
| Vomiting everything | 151 (9.6) | 38 (8.9) | 113 (10.0) | 0.520 |
| Convulsions | 789 (50.3) | 260 (60.7) | 525 (46.3) | <0.001 |

**Supplementary Table 2.** Clinical characteristics and associations with in-hospital mortality among the Sepsis Cohort, stratified by malaria smear positive or smear negative. Six patients with unknown malaria smear status are omitted.

| **Characteristic** | **n Malaria positive** | **Survived (row %)** | **Died at HGT (row %)** | **p-value*** | **n Malaria negative** | **Survived (row %)** | **Died at HGT (row %)** | **p-value*** |
| --- | --- | --- | --- | --- | --- | --- | --- | --- |
| **Age Group** |  |  |  |  |  |  |  |  |
| Less than seven days | 7 | 5 (71.4) | 2 (28.6) | .7199 | 93 | 51 (54.8) | 42 (45.2) | .0230 |
| 7 – 28 days | 9 | 7 (77.8) | 2 (22.2) |  | 66 | 36 (54.5) | 30 (45.4) |  |
| 29 days – 11 months | 136 | 95 (69.9) | 41 (30.1) |  | 484 | 330 (68.2) | 154 (31.8) |  |
| 12 – 59 months | 276 | 207 (75.0) | 69 (25.0) |  | 492 | 320 (65.0) | 172 (35.0) |  |
| **Sex** |  |  |  |  |  |  |  |  |
| Male | 228 | 167 (73.3) | 61 (26.8) | .9526 | 618 | 391 (63.3) | 227 (36.7) | .1986 |
| Female | 200 | 147 (73.5) | 53 (26.5) |  | 517 | 346 (66.9) | 171 (33.1) |  |
| **Bamako Residence** |  |  |  |  |  |  |  |  |
| Resident | 186 | 136 (73.1) | 50 (26.9) | .9195 | 585 | 388 (66.3) | 197 (33.7) | .3112 |
| Non-resident | 242 | 178 (73.6) | 64 (26.4) |  | 550 | 349 (63.4) | 201 (36.6) |  |
| **SIRS Criteria** |  |  |  |  |  |  |  |  |
| Abnormal temperature | 209 | 148 (70.8) | 61 (29.2) | .2435 | 636 | 395 (62.1) | 241 (37.8) | .0242 |
| Abnormal heart rate for age | 164 | 122 (74.4) | 42 (25.6) | .7052 | 395 | 254 (64.3) | 141 (35.7) | .7452 |
| Tachypnea for age | 411 | 299 (72.7) | 112 (27.3) | .1569 | 1059 | 693 (65.4) | 366 (34.6) | .1831 |
| One or more danger signs | 349 | 252 (72.2) | 97 (27.8) | .2546 | 939 | 592 (63.0) | 347 (37.0) | .0035 |
| Irritable | 58 | 45 (77.6) | 13 (22.4) | .4341 | 201 | 145 (72.1) | 56 (27.9) | .0183 |
| Lethargic | 170 | 117 (68.8) | 53 (31.2) | .0845 | 419 | 239 (57.0) | 180 (43.0) | <.0001 |
| Unconscious | 33 | 21 (63.6) | 12 (36.4) | .1882 | 39 | 21 (53.8) | 18 (46.2) | .1398 |
| Unable to feed | 132 | 85 (64.4) | 47 (35.6) | .0051 | 383 | 203 (53.0) | 180 (47.0) | <.0001 |
| Vomiting everything | 38 | 29 (76.3) | 9 (23.7) | .6664 | 113 | 71 (62.8) | 42 (37.2) | .6217 |
| Convulsions | 260 | 197 (75.8) | 63 (24.2) | .1615 | 525 | 334 (63.6) | 207 (36.4) | .2121 |
| **Diagnosis at admission** |  |  |  |  |  |  |  |  |
| Meningitis | 202 | 144 (71.3) | 58 (28.7) | .3580 | 553 | 347 (62.7) | 206 (37.3) | .1326 |
| Pneumonia | 80 | 61 (76.3) | 19 (23.8) | .5173 | 337 | 233 (69.1) | 104 (30.9) | .0537 |
| **Status prior to HGT** |  |  |  |  |  |  |  |  |
| Received antibiotics before HGT | 213 | 145 (68.1) | 68 (31.9) | .0137 | 583 | 375 (64.3) | 208 (35.7) | .6573 |
| Referred from healthcare facility† | 212 | 153 (67.7) | 59 (27.8) | .1401 | 719 | 449 (62.4) | 270 (37.6) | .0345 |
| Hospitalized before HGT† | 54 | 37 (68.5) | 17 (31.5) | .4880 | 186 | 108 (58.1) | 78 (41.9) | .1516 |
| **Antibiotics** |  |  |  |  |  |  |  |  |
| Received antibiotics at HGT | 316 | 226 (71.5) | 90 (28.5) | .1468 | 1033 | 662 (64.1) | 371 (35.9) | .0565 |
| Antibiotics <1 hour after admission‡ | 97 | 72 (74.2) | 25 (25.8) | .1231 | 506 | 325 (64.2) | 181 (35.8) | .1289 |
| Antibiotics 1-3 hours after admission | 150 | 108 (72.0) | 42 (28.0) |  | 379 | 234 (61.7) | 145 (38.3) |  |
| Antibiotics >3 hours after admission | 57 | 35 (61.4) | 22 (38.6) |  | 140 | 96 (68.6) | 44 (31.4) |  |
| No antibiotics | 112 | 88 (78.6) | 24 (21.4) |  | 101 | 74 (73.3) | 27 (26.7) |  |
| **Fluid Therapy** |  |  |  |  |  |  |  |  |
| Received fluids | 266 | 183 (68.8) | 83 (31.2) | .0062 | 608 | 377 (62.0) | 231 (38.0) | .0264 |
| Fluids <1 hour after admission | 75 | 54 (72.0) | 21 (28.0) | .0100 | 298 | 180 (60.4) | 118 (39.6) | .0161 |
| Fluids 1-3 hours after admission | 129 | 93 (72.1) | 36 (27.9) |  | 218 | 130 (59.6) | 88 (40.4) |  |
| Fluids >3 hours after admission | 58 | 34 (58.6) | 24 (41.4) |  | 91 | 66 (72.5) | 25 (27.5) |  |
| No fluids | 162 | 131 (80.9) | 31 (19.1) |  | 527 | 360 (68.3) | 167 (31.7) |  |
| Received maintenance fluids | 254 | 174 (68.5) | 80 (31.5) | .0208 | 586 | 364 (62.1) | 222 (37.9) | .0815 |
| Received bolus fluids | 12 | 9 (75.0) | 3 (25.0) |  | 22 | 13 (59.1) | 9 (40.9) |  |
| **Oxygen Therapy** |  |  |  |  |  |  |  |  |
| Received oxygen therapy | 91 | 53 (58.2) | 38 (41.8) | .0002 | 360 | 197 (54.7) | 163 (45.3) | <.0001 |
| Oxygen <1 hour after admission | 63 | 37 (58.7) | 26 (41.3) | .0036 | 267 | 144 (53.9) | 123 (46.1) | <.0001 |
| Oxygen 1-3 hours after admission | 14 | 8 (57.1) | 6 (42.9) |  | 59 | 32 (54.2) | 27 (45.8) |  |
| Oxygen >3 hours after admission | 14 | 8 (57.1) | 6 (42.9) |  | 34 | 21 (61.8) | 13 (38.2) |  |
| No oxygen therapy | 337 | 261 (77.4) | 76 (22.6) |  | 775 | 540 (69.7) | 235 (30.3) |  |
| Among children for which oxygen therapy was indicated¶ |  |  |  |  |  |  |  |  |
| Received oxygen therapy | 49 | 26 (53.1) | 23 (46.9) | .0005 | 238 | 138 (58.0) | 100 (42.0) | .2928 |
| No oxygen therapy | 31 | 28 (90.3) | 3 (9.7) |  | 106 | 55 (51.9) | 51 (48.1) |  |
| **Microbiology** |  |  |  |  |  |  |  |  |
| Culture positive | 22 | 8 (36.4) | 14 (63.6) | <.0001 | 141 | 67 (47.5) | 74 (52.5) | <.0001 |
| Culture negative | 406 | 306 (75.4) | 100 (24.6) |  | 994 | 670 (67.4) | 324 (32.6) |  |
| Gram-Positive Bacteria | 8 | 1 (12.5) | 7 (87.5) | <.0001 | 56 | 32 (57.1) | 24 (42.9) | .2102 |
| *Enterococcus* | 2 | 0 (0.0) | 2 (100.0) | - | 8 | 2 (25.0) | 6 (75.0) | - |
| *S. aureus* | 3 | 1 (33.3) | 2 (66.7) | - | 26 | 19 (73.1) | 7 (26.9) | .3787 |
| *S. pneumoniae* | 3 | 0 (0.0) | 3 (100.0) | - | 19 | 9 (47.4) | 10 (52.6) | .1056 |
| Group A *Streptococcus* |  |  |  |  | 2 | 1 (50.0) | 1 (50.0) | - |
| *Streptococcus* spp. |  |  |  |  | 2 | 2 (100.0) | 0 (0.0) | - |
| Gram-Negative Bacteria | 14 | 7 (50.0) | 7 (50.0) | .0443 | 83 | 37 (44.6) | 46 (55.4) | <.0001 |
| Gram-Negative Enterobacterales | 8 | 3 (37.5) | 5 (62.5) | .0205 | 52 | 21 (40.4) | 31 (59.6) | .0001 |
| *Enterobacter* spp. | 2 | 1 (50.0) | 1 (50.0) | - | 5 | 3 (60.0) | 2 (40.0) | - |
| *Escherichia* spp. | 2 | 0 (0.0) | 2 (100.0) | - | 11 | 4 (36.4) | 7 (63.6) | .0460 |
| *K. pneumoniae* | 3 | 1 (33.3) | 2 (66.7) | - | 22 | 4 (18.2) | 18 (81.8) | <.0001 |
| *Klebsiella* spp. |  |  |  |  | 1 | 0 (0.0) | 1 (100.0) | - |
| *Pantoea* spp. |  |  |  |  | 9 | 5 (55.6) | 4 (44.4) | .5539 |
| *Proteus* spp. |  |  |  |  | 1 | 0 (0.0) | 1 (100.0) | - |
| *Salmonella* Enterica nontyphoidal (NTS) | 1 | 1 (100.0) | 0 (0.0) | - | 3 | 3 (100.0) | 0 (0.0) | - |
| *Salmonella* Typhi |  |  |  |  | 2 | 2 (100.0) | 2 (0.0) | - |
| Gram-Negative Non-Enterobacterales | 6 | 4 (66.7) | 2 (33.3) | - | 32 | 17 (53.1) | 15 (46.9) | .1556 |
| *Acinetobacter* spp. | 2 | 2 (100.0) | 0 (0.0) | - | 4 | 2 (50.0) | 2 (50.0) | - |
| *Aeromonas* spp. |  |  |  |  | 1 | 1 (100.0) | 0 (0.0) | - |
| Nonfermenting gram-negative bacilli |  |  |  |  | 3 | 2 (66.7) | 1 (33.3) | - |
| *Burkholderia* spp. |  |  |  |  | 2 | 2 (100.0) | 0 (0.0) | - |
| *Chryseobacterium* spp. |  |  |  |  | 0 | 0 (100.0) | 1 (100.0) | - |
| *Haemphilus influenzae* type b | 3 | 2 (66.7) | 1 (33.3) | - | 7 | 7 (100.0) | 0 (0.0) | - |
| *Haemophilus influenzae* non-typable |  |  |  |  | 1 | 1 (100.0) | 0 (0.0) | - |
| *P. aeruginosa* | 1 | 0 (0.0) | 1 (100.0) | - | 13 | 2 (15.4) | 11 (84.6) | .0002 |
| Yeast | 1 | 0 (0.0) | 1 (100.0) | - | 2 | 0 (0.0) | 2 (100.0) | - |
| **Malnutrition** |  |  |  |  |  |  |  |  |
| Severe Acute Malnutrition | 153 | 102 (66.7) | 51 (33.3) | .0630 | 426 | 259 (60.8) | 167 (39.2) | .0492 |
| Moderate Acute Malnutrition | 77 | 55 (71.4) | 22 (28.6) |  | 206 | 143 (69.4) | 63 (30.6) |  |
| No Malnutrition | 171 | 135 (78.9) | 36 (21.1) |  | 419 | 285 (68.0) | 134 (32.0) |  |
| Unknown malnutrition status | 27 | 22 (81.5) | 5 (18.5) |  | 84 | 50 (59.2) | 34 (40.5) |  |
| Weight-for-height Z-score (WHZ) |  |  |  |  |  |  |  |  |
| WHZ < -3 | 117 | 81 (69.2) | 36 (30.8) | .3558 | 362 | 218 (60.2) | 144 (39.8) | .0136 |
| -3 ≤ WHZ < -2 | 67 | 46 (68.7) | 21 (31.3) |  | 152 | 110 (72.4) | 42 (27.6) |  |
| WHZ ≥ -2 | 215 | 164 (76.3) | 51 (23.7) |  | 529 | 356 (67.3) | 173 (32.7) |  |
| Unknown WHZ | 29 | 23 (79.3) | 6 (20.7) |  | 92 | 53 (57.6) | 39 (42.4) |  |
| Middle Upper Arm Circumference (MUAC) (among children aged ≥ 6 months, n = 1108) | | | | | | | | |
| MUAC <11.5 cm | 83 | 51 (61.4) | 32 (38.6) | .0273 | 168 | 104 (61.9) | 64 (38.1) | .8190 |
| 11.5 cm ≤ MUAC <12.5 cm | 52 | 39 (75.0) | 13 (25.0) |  | 194 | 129 (66.5) | 65 (33.5) |  |
| MUAC ≥12.5 cm | 159 | 126 (79.2) | 33 (20.8) |  | 362 | 235 (64.9) | 127 (35.1) |  |
| Unknown | 46 | 35 (76.1) | 11 (23.9) |  | 42 | 28 (66.7) | 14 (33.3) |  |
| Bilateral pitting edema | 1 | 0 (0.0) | 1 (100.0) | - | 2 | 0 (0.0) | 2 (100.0) | - |
| **Severe Sepsis Indicators** |  |  |  |  |  |  |  |  |
| Met criteria for severe sepsis | 299 | 211 (70.6) | 88 (29.4) | .0464 | 851 | 500 (58.8) | 351 (41.2) | <.0001 |
| Blantyre Coma Score < 5 | 213 | 149 (70.0) | 64 (30.0) | .2374 | 671 | 379 (56.5) | 292 (43.5) | <.0001 |
| Oxygen saturation < 90% | 74 | 49 (66.2) | 25 (33.8) | .1850 | 339 | 191 (56.3) | 148 (43.7) | <.0001 |
| Acute oliguria | 89 | 60 (67.4) | 29 (32.6) | .1537 | 133 | 80 (60.2) | 53 (39.8) | .2185 |
| Signs of respiratory distress | 11 | 8 (72.7) | 3 (27.3) | - | 4 | 3 (75.0) | 1 (25.0) | - |
| Peripheral or central cyanosis | 4 | 3 (75.0) | 1 (25.0) | - | 5 | 1 (20.0) | 4 (80.0) | - |
| Decreased capillary refill | 2 | 2 (100.0) | 0 (0) | - | 6 | 3 (50.0) | 3 (50.0) | - |
| Petechiae, ecchymoses, or purpura | 3 | 3 (100.0) | 0 (0) | - | 0 | - | - | - |
| **Length of Stay** |  |  |  |  |  |  |  |  |
| <1 day | 36 | 15 (41.7) | 21 (58.3) | <.0001 | 116 | 18 (15.5) | 98 (84.5) | <.0001 |
| 1 day | 78 | 31 (39.7) | 47 (60.3) |  | 187 | 53 (28.3) | 134 (71.7) |  |
| 2 days | 51 | 29 (56.9) | 22 (43.1) |  | 112 | 56 (50.0) | 56 (50.0) |  |
| 3 days | 44 | 36 (81.8) | 8 (18.2) |  | 100 | 65 (65.0) | 35 (35.0) |  |
| 4 – 5 days | 65 | 63 (96.9) | 2 (3.1) |  | 169 | 143 (84.6) | 26 (15.4) |  |
| 6 – 7 days | 53 | 47 (88.7) | 6 (11.3) |  | 146 | 125 (85.6) | 21 (14.4) |  |
| 8 – 10 days | 46 | 44 (95.7) | 2 (4.3) |  | 111 | 102 (91.9) | 9 (8.1) |  |
| 11 – 14 days | 18 | 17 (94.4) | 1 (5.6) |  | 83 | 75 (90.4) | 8 (9.6) |  |
| 15+ days | 37 | 32 (86.5) | 5 (13.5) |  | 111 | 100 (90.1) | 11 (9.9) |  |
| **Total** | **428** | **314 (73.4)** | **114 (26.6)** |  | **1135** | **737 (64.9)** | **398 (35.1)** |  |

* p-values were calculated using Pearson’s chi-square test for categorical variables. p-values were not reported for comparisons involving extremely sparse categories (e.g., rare binary variables or low-frequency pathogen groups), where results were not considered interpretable due to limited sample size; these are indicated as not estimable (-).

† Among patients who were assessed for referral from other healthcare facility prior to arrival at HGT (n = 1,428)

‡ Time of therapy administration is unknown for some patients; these patients are included in the binary therapy variable but omitted from the timeliness strata

¶ Among patients for which oxygen therapy is indicated, which includes patients who have an oxygen saturation on room air ≤ 90%, have chest indrawing, or have difficulty breathing

**Supplementary Table 3.** Antibiotic susceptibility and in-hospital mortality by antibiotic administered among 156 culture-positive children who received antibiotics.

| **Antibiotic** | **Received, n** | **Susceptibility assessed n (% of received)** | **Sensitive** | | | **Resistant or Intermediate** | | |
| --- | --- | --- | --- | --- | --- | --- | --- | --- |
|  |  |  | **n (% of assessed)** | **Survived n (%)** | **Died n (%)** | **n (% of assessed)** | **Survived n (%)** | **Died n (%)** |
| Gentamicin | 115 | 71 (61.7) | 31 (43.7) | 16 (51.6) | 15 (48.4) | 40 (56.3) | 17 (42.5) | 23 (57.5) |
| Ceftriaxone | 82 | 31 (37.8) | 8 (25.8) | 8 (100) | 0 (0) | 23 (74.2) | 6 (28.6) | 17 (73.9) |
| Cefotaxime | 58 | 12 (20.7) | 4 (33.3) | 3 (75.0) | 1 (25.0) | 8 (66.7) | 4 (50.0) | 4 (50.0) |
| Amoxicillin/ Clavulanic Acid | 14 | 1 (7.1) | 0 (0) | - | - | 1 (100) | 0 (0) | 1 (100) |
| Ciprofloxacin | 3 | 3 (100) | 2 (66.7) | 0 (0) | 2 (100) | 1 (33.3) | 0 (0) | 1 (100) |
| Other antibiotic* | 35 | 0 (0) | - | - | - | - | - | - |

*Other antibiotics include amoxicillin (2), amikacine (1), vancomycin (16), metronidazole (17), rifamycin (1), and josamycin (1). Patients often received multiple antibiotics, and none of these listed were assessed for resistance.
